# Single‐cell RNA sequencing to explore cancer‐associated fibroblasts heterogeneity: “Single” vision for “heterogeneous” environment

**DOI:** 10.1111/cpr.13592

**Published:** 2023-12-29

**Authors:** Xiangjian Zhang, Ruiqiu Zhu, Die Yu, Juan Wang, Yuxiang Yan, Ke Xu

**Affiliations:** ^1^ The Dingli Clinical College of Wenzhou Medical University Wenzhou Zhejiang China; ^2^ Department of Surgical Oncology Wenzhou Central Hospital Wenzhou Zhejiang China; ^3^ The Second Affiliated Hospital of Shanghai University Wenzhou Zhejiang China; ^4^ Interventional Cancer Institute of Chinese Integrative Medicine Putuo Hospital, Shanghai University of Traditional Chinese Medicine Shanghai China; ^5^ School of Medicine Shanghai University Shanghai China; ^6^ Institute of Translational Medicine Shanghai University Shanghai China; ^7^ Organoid Research Center Shanghai University Shanghai China; ^8^ Wenzhou Institute of Shanghai University Wenzhou China

## Abstract

Cancer‐associated fibroblasts (CAFs), a phenotypically and functionally heterogeneous stromal cell, are one of the most important components of the tumour microenvironment. Previous studies have consolidated it as a promising target against cancer. However, variable therapeutic efficacy—both protumor and antitumor effects have been observed not least owing to the strong heterogeneity of CAFs. Over the past 10 years, advances in single‐cell RNA sequencing (scRNA‐seq) technologies had a dramatic effect on biomedical research, enabling the analysis of single cell transcriptomes with unprecedented resolution and throughput. Specifically, scRNA‐seq facilitates our understanding of the complexity and heterogeneity of diverse CAF subtypes. In this review, we discuss the up‐to‐date knowledge about CAF heterogeneity with a focus on scRNA‐seq perspective to investigate the emerging strategies for integrating multimodal single‐cell platforms. Furthermore, we summarized the clinical application of scRNA‐seq on CAF research. We believe that the comprehensive understanding of the heterogeneity of CAFs form different visions will generate innovative solutions to cancer therapy and achieve clinical applications.

## Graphical abstract

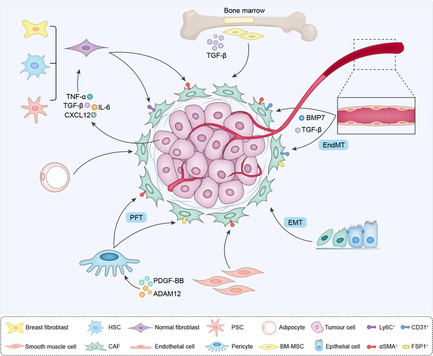

## 1 INTRODUCTION

Cancer is one of the leading causes of death in humans.^1^ Currently, tumour cells are the main target of cancer therapy. However, fatal metastasis, recurrence and refractory tumour microenvironment (TME) has always been the key problems that hinder the effectively targeted therapy response.^2^, ^3^ TME is a multicellular system primarily composed of tumour cells, cancer‐associated fibroblasts (CAFs), and immune cells including tumour‐infiltrating lymphocytes and macrophages, as well as extracellular matrix (ECM). Existing evidence reveals its critical roles in the development and progression of multiple malignancies.

CAFs are one of the most important components in the TME, which contribute to multiple aspects of tumorigenesis by degrading and remodelling ECM, secreting growth factors, cytokines and inducing anti‐tumour immune invasion.^4^ CAFs have shown high heterogeneity, which differ in their origin, phenotype and functions. CAFs can originate from resident tissue fibroblasts, bone marrow‐derived mesenchymal stem cell, adipocyte, endothelial cells and epithelial cells. In the aspects of functional heterogeneity, CAFs can generate and remodel the ECM and are the central players in the deposition of the ECM environment. Besides, highly heterogeneous CAFs play a role in tumorigenesis, negative regulation of anti‐tumour immunity, tumour invasion and metastasis by secreting some cytokines and growth factors. Consistently, distinct CAF subtypes are characterized by distinct markers and biphasic functions—cancer‐restraining and cancer‐promoting. Thus, understanding the heterogeneity will provide novel insights into how this might tangle the TME up in cancer and hence develop potential treatment strategies.

Traditional gene‐expression analysis techniques, such as quantitative PCR, microarray and bulk RNA sequencing, always analyse cell populations as a whole. These methods may have ignored cell populations with low abundance but crucial functions. The emerging of single‐cell RNA sequencing (scRNA‐seq) technology has begun to solve these limitations by analysing the transcriptome of every cell with unprecedented resolution and accuracy, and innovate our understanding of oncogenesis. The CAF research has also embraced this new technology, with an abundance of CAF scRNA‐seq studies reported in the last 10 years. In addition to identifying rare or new subpopulations of cells, scRNA‐seq enables cellular trajectory analysis on the basis of each cell transcriptome. Moreover, scRNA‐seq also enables cross‐profiling of single‐cell transcriptomes of multiple cell types, and thus, cell‐to‐cell communication can be predicted based on via ligand‐receptor interactions. Importantly, we can identify the specific targets of tumour promoting CAFs using scRNA‐seq, so as to construct individualized adjuvant therapy.

In this review, we will discuss the CAF heterogeneity, with a focus on how scRNA‐seq techniques transform our knowledge of CAF biology and tumorigenesis and summarize the clinical application of scRNA‐seq in CAFs. The comprehensive understanding of the heterogeneity of CAFs form traditional to scRNA‐seq perspective is expected to improve the gap in the CAFs field and accelerate the advancement of precision medicine.

## 2 CAFS HETEROGENEITY FROM TRADITIONAL TECHNOLOGY

### 2.1 Origin heterogeneity of CAFs

**FIGURE 1:**
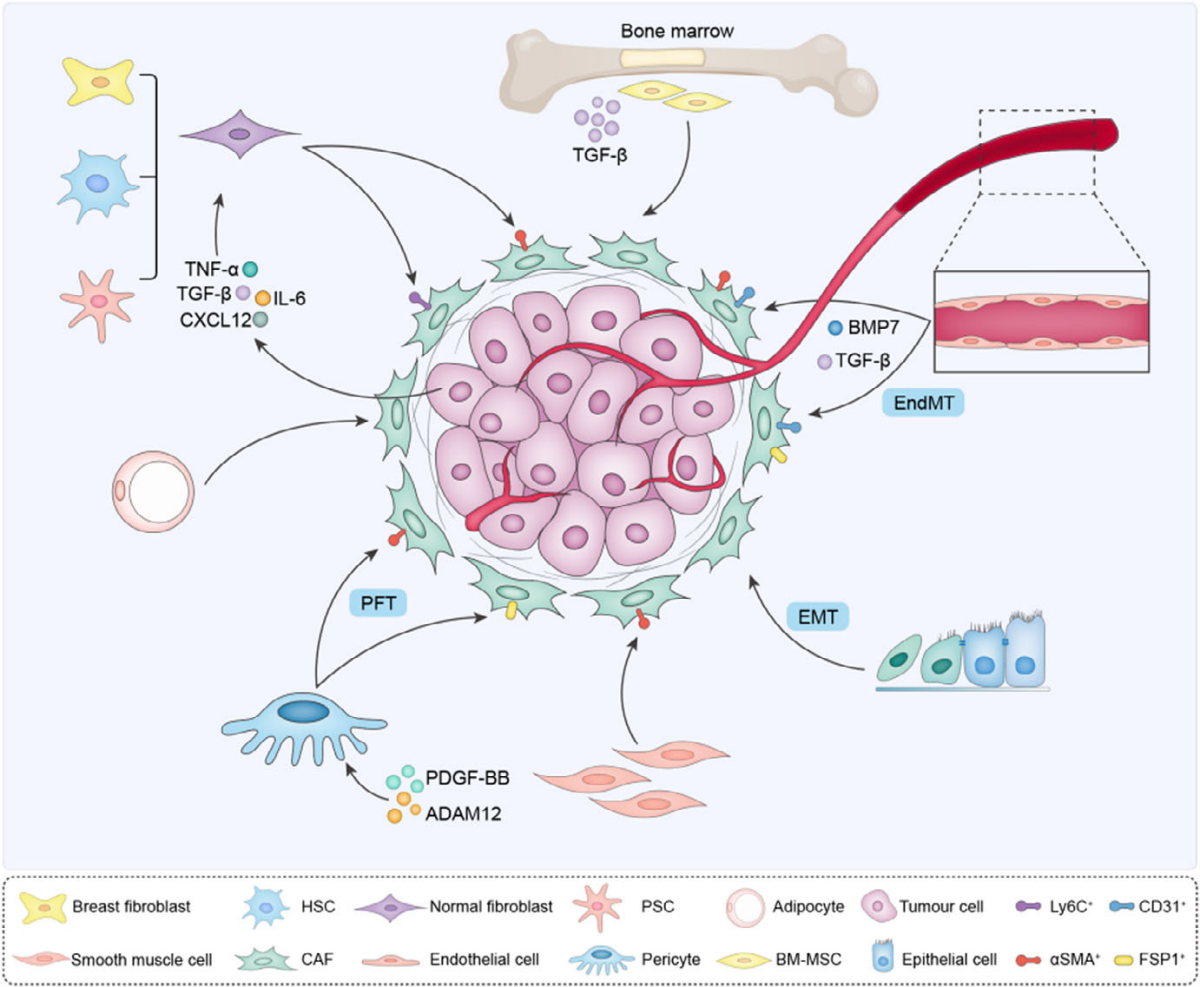
The origin heterogeneity of CAFs. Multiple types of cells, under certain conditions, may become tumour‐associated fibroblasts (CAFs). A variety of normal fibroblasts, including resident breast fibroblasts, pancreatic stellate cells (PSCs) and hepatic stellate cells (HSCs), bone marrow‐derived mesenchymal stem cells (BM‐MSCs) transformed into CAFs under the action of cytokines such as TGF‐β. Endothelial cells convert into CAFs through endothelial‐mesenchymal transition (EndMT), and epithelial cells differentiate toward CAFs through epithelial mesenchymal‐transition (EMT). Adipocytes, pericytes and smooth muscle cells can also be the source of CAFs.

Emerging evidence strongly suggests that CAFs originate from multiple cells,^5^ which might partially explained CAF heterogeneity in tumours (Figure 1).

Most commonly, CAFs can be recruited and activated from normal tissue fibroblasts.^6^, ^7^ Similar to wound‐healing, the formation of activated fibroblasts is mainly dependent on microenvironment cues. In a breast tumour xenograft model, resident human breast fibroblasts gradually transformed into CAFs under the stimulation of TGF‐β and stromal cell‐derived factor‐1 (SDF‐1), which initiate and maintain the conversion of fibroblasts into activated myofibroblasts (myCAFs) and cancer‐promoting phenotype during tumour progression.^6^ Consistently, fibroblasts in the pancreas and liver, such as stationary pancreatic stellate cells (PSCs) and hepatic stellate cells (HSCs), could also be activated and obtain myCAF and secretory phenotypes. This process mainly depends on paracrine factors, such as cytokines (IL‐1, IL‐6, IL‐8 and TNF‐α), growth factors (PDGF and TGF‐β), angiotensin II and reactive oxygen species,^8^, ^9^ highlighting the inducible potency of normal fibroblasts in different tissues.

CAFs can also be derived from endothelial cells through endothelium‐mesenchymal transformation (EndMT).^10^, ^11^, ^12^ The endothelium is a single layer of squamous cells, which provide the intracellular layer of blood vessels and lymphatic vessels. Current researches indicate that endothelial cell is a source of CAF types via EndMT.^13^, ^14^ TGF‐β stimulates the conversion of endothelial cells into CAFs, while bone morphogenic protein 7 (BMP7) maintains the endothelial fate,^15^ indicating that antiangiogenic treatment of tumours, such as TGF‐β inhibitors or BMP7 agonists, may function as potential targets for decreasing activated fibroblasts. CAFs derived from EndMT were identified as a unique cell population that co‐expressed the endothelial marker CD31 and mesenchymal marker FSP1 or α‐SMA.

Epithelial cells can acquire mesenchymal expression programs and convert into mesenchymal cells (epithelial‐mesenchymal transition, EMT).^16^ EMT has been proposed as an important source of CAFs involved in tumour invasiveness and metastasis. In vivo and in vitro studies demonstrate that carcinoma cells can obtain CAF marker, such as alpha smooth muscle actin (α‐SMA), fibroblast activation protein (FAP) and vimentin.^17^ Multiple EMT‐triggering signals have been revealed, notably TGF‐β, Wnt and PDGF.

Mesenchymal cells (MSCs) are other likely important origins of CAFs. Under the action of TGF‐β, bone marrow‐derived MSCs (BM‐MSCs), adipocyte‐derived stem cells and smooth muscle cells differentiate into CAFs, promoting the progression of tumours, such as B‐cell acute lymphoblastic leukaemia, glioma, breast, pancreatic, or gastric tumour.^18^, ^19^, ^20^ Moreover, pericytes transdifferentiate into CAFs via pericyte to fibroblast transition (PFT).^21^ ADAM12 (a disintegrant and metalloprotease 12) and PDGF‐BB promote pericytes to obtain CAF phenotype.^21^, ^22^ In line, targeting PDGF‐BB with imatinib blocks PFT, accompanied with reduced activated CAF components in tumours and delayed tumour growth and metastasis, suggesting that PFT inhibition would be an effective approach for cancer therapy.

In general, the original heterogeneity of CAFs is very complex. The difference in origins leads to the functional differences of CAFs to some extent. Meanwhile, CAFs from the same source will also produce functional heterogeneity due to different external factors that stimulate transformation and generate CAFs with different surface markers.

### 2.2 Surface biomarkers of CAFs

Activated CAFs are characterized by a handful of different biomarkers (Table 1), such as α‐SMA, FAP, platelet‐derived growth factor receptor (PDGFR) α/β, fibroblast specific protein 1 (FSP1, also known as S100A4), vimentin (VIM) and podoplanin (PDPN).^23^, ^24^ CAF markers may also be tightly associated with distinct functions in the context of TME. Notably, these biomarkers are not specific for CAFs, nor always expressed all of these putative markers.

**TABLE 1:** Summary of surface biomarkers of CAFs.

Biomarkers	Expression level in CAFs	Expression in other cell types	Functions	References
α‐SMA	Upregulated (downregulated in prostate cancer)	Normal fibroblasts, pericytes, smooth muscle cells, visceral smooth muscle cells and cardiomyocytes	Motor function, maintenance of the basic structure of fibroblasts, ECM tissue synthesis and collagen formation	^38^, ^39^, ^40^
FAP	Upregulated	Glucagon^+^ alpha cells, CD45^+^ cells	Inhibit T cell proliferation, muscle contraction, ECM remodelling and fibrogenesis	^30^, ^41^
PDGFR	Upregulated	Normal fibroblasts, pericytes, vascular smooth muscle cells, skeletal muscles and myocardium	Cancerous epithelia proliferation and metastasis, angiogenesis	^42^, ^43^
FSP1 (S100A4)	Upregulated	Normal fibroblasts, epithelial cells undergoing EMT and macrophages	Collagen production, tumour immune escape and invasion	^44^, ^45^
VIM	Upregulated	Fibroblasts, endothelial cells, lymphocytes and several specialized cells of the thymus and the brain	Cytoskeleton and cell motility	^46^
PDPN	Upregulated	Lymphatic endothelial cells (LEC)	Tumour cell migration and invasion	^47^, ^48^
CAV1	Upregulated or downregulated	Normal fibroblasts, adipocytes, Endothelial cells	Tumour progression was promoted by remodelling tumour matrix	^49^, ^50^

#### 2.2.1 α‐SMA

α‐SMA, also known as smooth muscle aorto‐actin (ACTA2), is a member of the actin family and plays an important role in maintaining structural integrity and cell movement. α‐SMA has become one of the preferred markers to identify CAF populations.^25^ In addition, α‐SMA has also been identified as an important prognostic factor. In breast and colon cancer patients, high level of α‐SMA^+^ fibroblasts in cancer tissue was associated with reduced overall survival.^26^ Of note, α‐SMA is also commonly expressed in pericytes and vascular muscle cells. Therefore, the effect of the interfering cells must be considered when analysing α‐SMA^+^ cells.^27^, ^28^

#### 2.2.2 FAP

FAP is a common type II intact membrane protein belonging to the membrane‐bound serine protease family.^29^ In general, FAP expression is indeed low in normal adult tissues, but becomes upregulated in almost all carcinomas.^30^ Studies have revealed the relevance of FAP with higher tumour grade, metastasis and worse overall survival, such as in breast invasive ductal carcinoma, colon cancer and gastric cancer.^31^, ^32^, ^33^ Functionally, FAP^+^CAFs promote tumour immunosuppression, regulate fibroblast growth, ECM remodelling^34^, ^35^, ^36^ and induce angiogenesis in the tumour microenvironment.^37^ Intriguingly, a recent study confirmed the existence of heterogenous.

FAP‐expressing stromal cells in murine breast cancer.^41^ A FAP^+^PDPN^+^ population of CAFs, which are located at the outer edge of the tumour, contributes to inhibiting the proliferation of T cells, while FAP^+^PDPN^−^ population appear not to be immunosuppressive. Besides, FAP has also been identified in non‐CAF components, such as macrophages, pericytes. Even in CAFs, not all CAFs express FAP. For example, only CAF‐A cells expresses FAP in human colorectal tumours.^51^

#### 2.2.3 PDGFRα/β

PDGFR, a tyrosine kinase receptor, is usually used as a general marker of fibroblasts in a wide variety of tumour types, such as glioma, prostate and ovarian cancer.^52^ Platelet‐derived factors (PDGFs) mediate biological effects of CAFs by interacting with PDGFR. There are two forms: PDGFRα and PDGFRβ. Interestingly, these two receptors seem to have diverse functions. PDGFRα^−^PDGFRβ^+^ CAFs in breast cancer display increased expression of ECM remodelling enzymes and TGF‐β ligands, and this type of CAFs promotes the occurrence of invasive cancer. Whereas, PDGFRα^+^PDGFRβ^−^CAFs are mainly responsible to secrete inflammatory silver to promote the EMT process of tumour cells.^53^ Therefore, it is incredible to define CAF subset function by a single PDGFR. What's more, PDGFR is present in all types of cells. Therefore, it can only be used as a supporting indicator.

#### 2.2.4 FSP1

FSP1 is expressed relatively specifically on fibroblasts, although varied in different CAF subgroups.^14^ FSP1^+^CAFs appear to prevent epithelial malignancy via collagen production.^54^ Moreover, a large number of FSP1^+^CAFs cluster in the metastatic matrix, indicative of a role in tumour metastasis.^55^ Intriguingly, more recent studies have also identified the high expression of FSP1 in immune suppressive T and myeloid cells, which suppress the immune response, and deleting FSP1 in non‐cancer cells elevated survival rate and change immune landscape, supporting the pro‐tumour effect of FSP1.^56^ These conflicting findings may suggest the different function of FSP1 in different cell types.

#### 2.2.5 Other markers

VIM is also a key marker in CAFs associated with tumour metastasis,^57^ but the underlying mechanism remains to be clarified. In addition to CAFs, populations of VIM^+^cells also exist in both macrophages and fat cells.^58^ Besides, PDPN has been widely used as a targeted marker of CAFs. PDPN expression is significantly upregulated in inflammation and cancer, and function as a good predictor of tumour malignancy and a target for biological therapy, but it is also expressed in macrophages, T cells and epithelial cells.^59^ Moreover, S100A4 and PDPN seems to be negatively correlated to some extent.^60^, ^61^, ^62^ Caveolin (CAV1), a member of the stenting protein family, is differentially expressed in CAFs, and loss of CAV1 in breast and prostate CAFs lead to tumour invasion and progression, while high expression of CAV1 in CAFs could also facilitate tumour invasion via ECM remodelling,^49^, ^50^, ^63^ suggesting the specific functions in different TME. For example, Silencing of CAV1 in fibroblasts results in increased tumour growth rate and chemoresistance in a human pancreatic cancer model.^64^ CAV1 plays a central role in radioresistance‐mediated tumour‐stroma interactions in advanced prostate cancer (PCa), CAV1‐deficient endothelial cells increased growth delay of PCa cell after radiation treatment.^65^ Additionally, CAV1 expression confers a proliferative advantage in lung adenocarcinoma cells, fostering increased tumour aggressiveness.^66^

Although great advances have been made in the past studies, there are still several unsolved problems. First, it is rough for a single biomarker to screen out the CAF subpopulations from the complex TME. Second, the CAF subsets identified by the existing biomarker can only one‐sidedly represent a class of CAF cells, which will have great limitations if translate to clinical treatment. For example, ACTA2^+^CAFs are essential for restricting tumour invasion and progression in mouse pancreatic ductal adenocarcinoma models,^67^ yet promoting tumour progression in some cases, at least partially via influencing TME remodelling.^68^ Thus, single targeting ACTA2 for anti‐tumour therapy resulted in variable efficacy, and possibly opposing. Due to poor technical means, we haven't thoroughly dug out useful information. It is a bold guess that some biomarker‐positive CAFs may account for only a small part of the CAF populations, but they have a strong tumour‐promoting function, and their specific biomarkers will be masked and undetectable using traditional sequencing. Therefore, CAFs cannot be well classified either in terms of their origin heterogeneity or surface markers.^67^, ^69^ Fortunately, emerging high‐throughput analysis of scRNA‐seq can solve this problem to some extent.

## 3 SINGLE‐CELL RNA SEQUENCING TECHNIQUES

**FIGURE 2:**
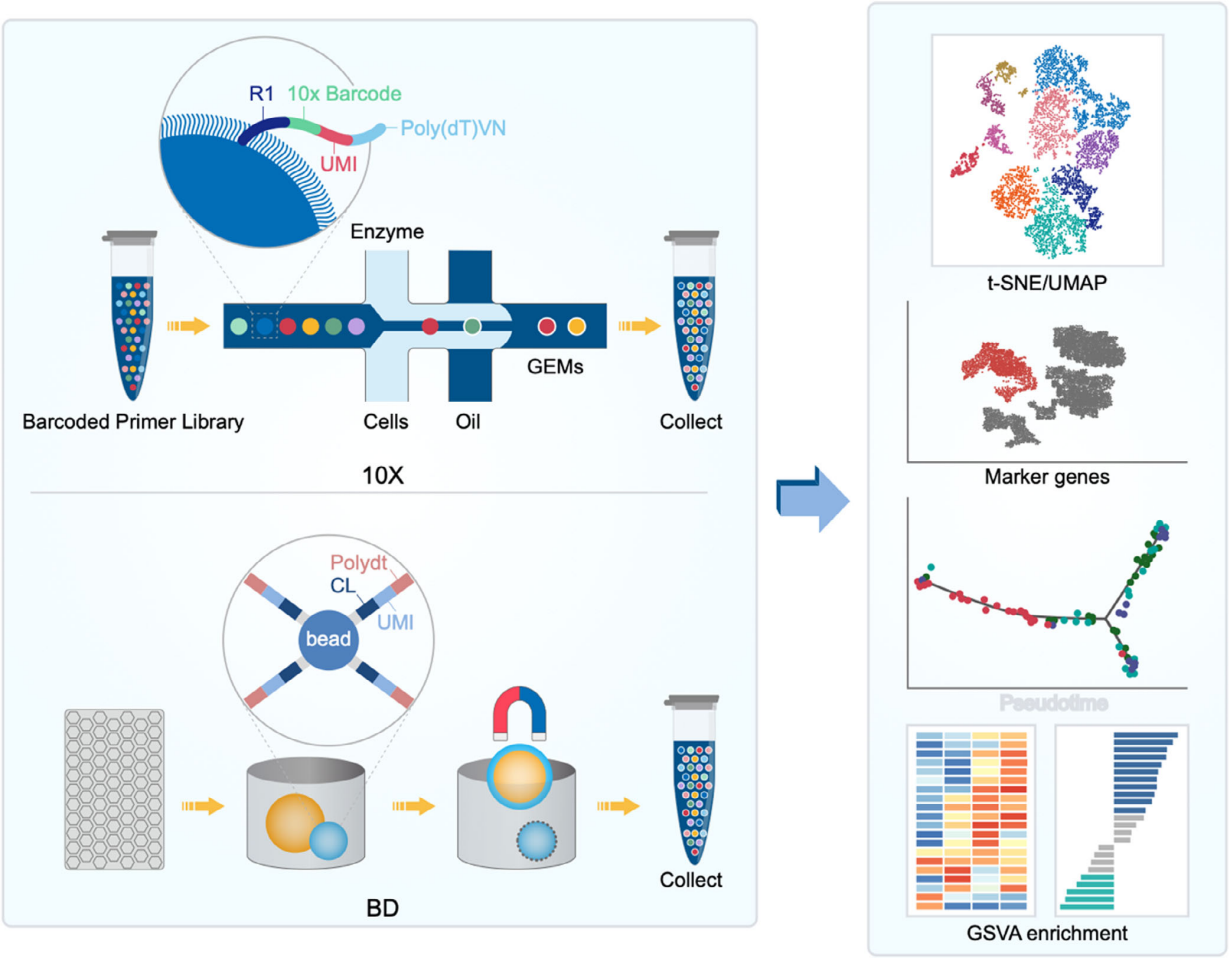
ScRNA‐seq platform and data processing. The 10× platform separates and specifically identifies individual cells through a special oil‐in‐water structure, forming GEMs (oil‐in‐water Microsystems). After GEMs is formed, the cell will be broken, the gel beads will be dissolved automatically and a large amount of barcode sequence will be released, and then the mRNA reverse transcription will produce cDNA, thus constructing the standard sequencing library. The BD platform separates individual cells through the structure of the honeycomb plate. Excess beads bind to individual cells to further break down cells and construct cDNA libraries. t‐SNE/UMAP analysis was performed to identify the cell subtypes. Marker genes of various subtypes can also be observed. In several subtypes, molecular differentiation can be observed through Pseudotime. GSVA enrichment analysis may reveal the functional differences of different subtypes.

It is well known that cells within a population are also highly dynamic and specific, including the process of differentiation, which involves changes in gene expression.^70^, ^71^, ^72^ Under certain conditions, some small number of cells may have an important role in pathological conditions, such as tumour progression.^73^ Thus, specifically targeting the key cell types would obtain favourable outcomes with minimal side effects. However, traditional research methods always discuss the characteristics of this group of cells as a whole, and the smaller cell subsets with critical functions will be overshadowed by the large number of cells. The advent of single‐cell sequencing has substantially revolutionized knowledge of certain cell lines. Single‐cell level provides us with a platform for detailed analysis of each cell subgroup. scRNA‐seq platform and data processing was summarized in Figure 2.

The principle of single‐cell transcriptome sequencing is separating the individual cells of a trace of mRNA in high‐throughput sequencing. Efficient amplification with single‐cell transcriptome sequencing can effectively solve the problems of conventional RNA‐seq of cells within the transcriptome, including heterogeneity, by identifying rare cell types and obtaining an in‐depth understanding of molecular mechanisms. The most common form of single‐cell transcriptome sequencing is 10× Platform. The microreaction system of oil‐coated water is used to distinguish different cells by cell barcode and UMI, thus obtaining the gene expression profile at the single‐cell level. In addition, there is a C1 system (Fluidigm), which can obtain all the transcriptome information at the single‐cell level through microfluidics technology. The BD Rhapsody system uses honeycomb panel technology, which adds excess beads to each cell for unique identification. After cell lysis, the released mRNA is used for single‐cell reverse transcription amplification and library construction. In addition, compared with the 10× platform, the BD platform has lower requirements for cell activity. Therefore, freezed tissues can also be used in this system, which gives us much time to carry out the preliminary sequencing work. In summary, different platforms provide different cell capture methods, but the main purpose is to isolate a single cell to obtain its genetic information for amplification and library building and to prepare for subsequent analyses.

After obtaining high‐quality sequencing data, single‐cell sequencing has a series of analytical processes, including presenting our data through quality control, alignment, read quantification, expression matrix filtering, normalization and visualization. With the development of single‐cell sequencing, there are now a number of popular tools that can cover the entire process of analysis, as well as single‐cell sequencing databases for our reference.

Through the dimension reduction analysis of tSNE and UMP, we can clearly observe the subtypes of the samples tested and visualize the results by clustering analysis. Markers for each subtype can also be displayed in a variety of ways. By using pseudotime analysis, we can observe the potential molecular differentiation between cells. Different genes hint functional differences and the enrichment of different signalling pathways.

ScRNA‐seq has been increasingly used in biology and physiology. Single‐cell sequencing, combined with other techniques, could be used not only to map the trajectory of cell differentiation but also to reveal the cell subtypes of various cell types. For example, transcriptome analysis revealed previously unknown cell types and subtypes in normal and diseased livers and hearts.^74^, ^75^, ^76^ Single‐cell resolution provided a thorough understanding of the spatiotemporal dynamics of the development of embryonic macrophages.^77^ In a study of the novel coronavirus, single‐cell transcriptome analysis constructs a cellular map of the peripheral immune in response to severe COVID‐19.^78^ Additionally, through single‐cell sequencing technology, a great breakthrough has also been made in astrocyte subtype classification and cell type identification.^79^, ^80^ Similarly, we believe that the proper use of single‐cell sequencing technology can increase the clarity of CAF subtype classification.

## 4 CAF HETEROGENEITY FROM SINGLE‐CELL RNA SEQUENCING

### 4.1 CAF differentiation

**FIGURE 3:**
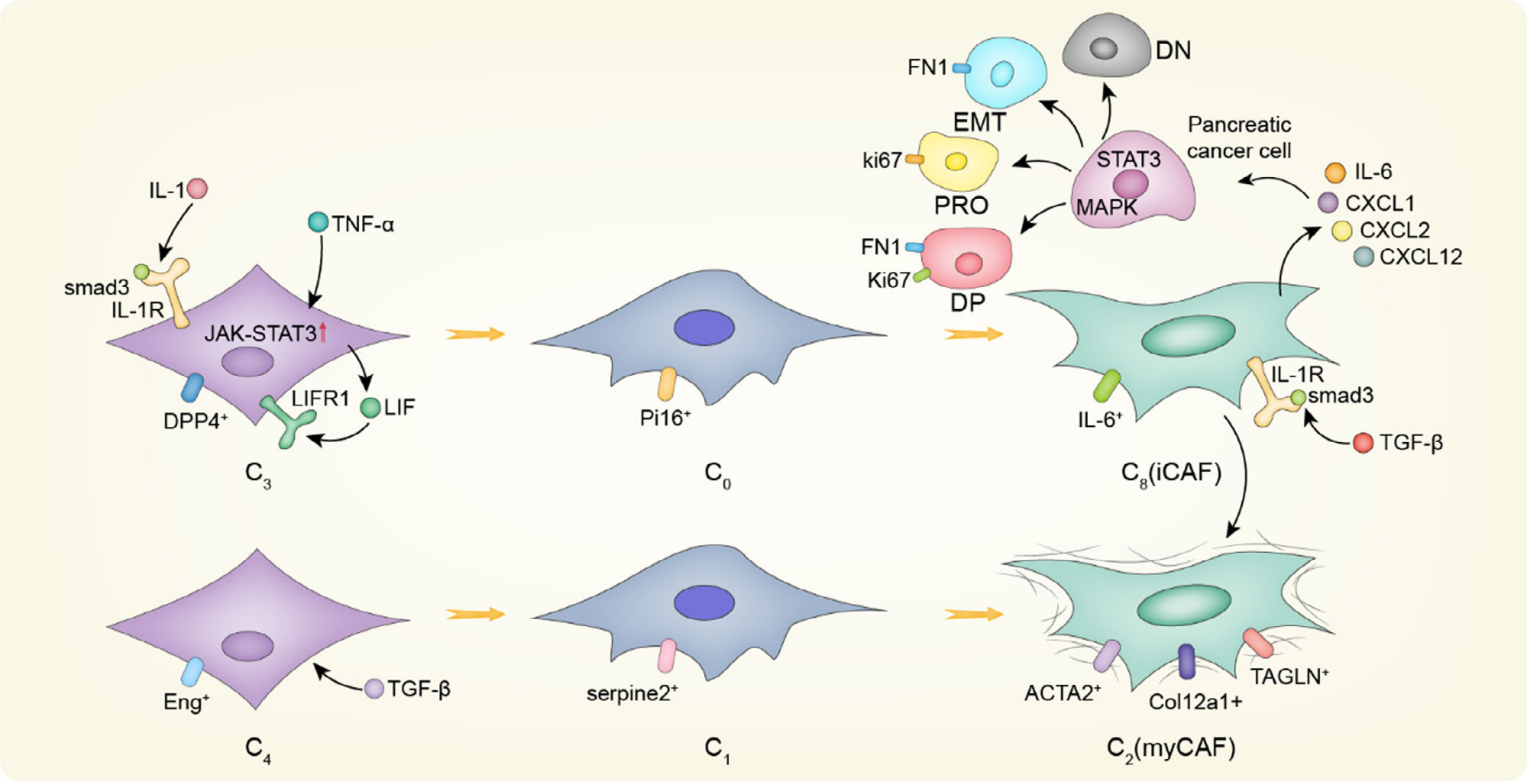
Molecular differentiation model of CAFs in PDAC. Normal fibroblast C3 subtype initiates the differentiation process under the action of IL‐1 and TNF‐α signals, and the C0 subtype becomes the transition state and finally differentiates into C8 (iCAF) with secretion function, which secretes IL‐6, CXCL1, CXCL2 and other cytokines. Under the action of these cytokines, pancreatic cancer cells progress into four subtypes: Ki67^+^ (characterized by proliferation, PRO), FN1^+^(characterized by EMT), double negative (DN, Ki67^−^FN1^−^) and double positive (DP, Ki67^+^FN1^+^). Normal fibroblast C4 subtype starts differentiation under the action of TGF‐β, and give rise to C1 subtype, which finally differentiates into C2 (myCAF) with drastic expressions of fibrillar collagens, while iCAF can also be directly transformed into myCAF under the action of TGF‐β.

Before the emergence of single‐cell sequencing, the evolution process of CAFs has always been a mystery. Revealing the differentiation process of CAFs will provide new insights into CAF subtypes and guide clinical therapy. CAF differentiation models have been summarized in Figure 3. The existence of inflammatory CAFs (iCAFs) and myofbroblastic CAFs (myCAFs) subtypes in pancreatic ductal adenocarcinoma (PDAC) was first proposed by Daniel Ohlund.^27^ myCAFs express genes associated with collagen formation, ECM remodelling and smooth muscle contraction, such as α‐SMA, and are located immediately neighbouring to tumour cells; iCAFs are located distantly from neoplastic cells, which display less α‐SMA, but secrete IL‐6 and other inflammatory factors. According to the results of scRNA‐seq and functional analyses, TGF‐β and IL‐1/STAT3 signalling have been identified as the main pathways responsible for the formation and maintenance of myCAFs and iCAFs, respectively, in mouse and human PDAC and organ‐like models.^38^ A small number of α‐SMA and P‐STAT3 double‐positive cells were identified by immunofluorescence, indicating the presence of other CAF subtypes or intermediate states between the iCAF and myCAF phenotypes, thus supporting the potential plasticity of these two cell subtypes in vivo. Coincidentally, Dominguez CX et al. demonstrated the existence of intermediate state of CAFs by first defining two independent normal tissue fibroblasts (ntFibs) as C3 and C4.^35^ These ntFibs displayed different functions, with C4 providing more structural support but C3 showing more immunoregulatory potential. Five CAF subtypes of C0, C1, C2, C8 and C9 were identified by analysing the tumours with different pathological degrees. Through time series analysis and characteristic gene comparison of each subgroup, it was revealed that in the background of PDAC, C3 and C4 ntFibs were driven to differentiate into CAFs by IL‐1 and TGF‐β, respectively, and there was an obvious differentiation process: C3 was differentiated into C0 and then further differentiated into C8 displaying iCAF‐like features with high level of IL‐6 and other inflammatory factors, while C4 was differentiated into C1 and then differentiated into C2, which resembles myCAF phenotype expressing high level of fibrillar collagens. Moreover, C8 and C2 increased with tumour progression and played a dominant role in late‐stage tumours, indicating the clinical relevance. Similar finding was also described in breast cancer with scRNA‐seq study of patient‐derived fibroblasts and CAFs in mice launched by Busch S et al.^81^ Normal fibroblasts were found to gradually lose their original characteristics and progressed into more CAF‐like phenotype that promote the development and drug resistance of cancer. Due to the small number of gene samples analysed or the limitation of technology, no complete match was found in patient samples when cross‐species analysis was conducted. However, both studies identified distinct but overlap gene profiles in CAFs, indicative of underlying hierarchical and differentiation process, and facilitate the discovery of specific marker and develop novel targets for anti‐CAF therapy.

Of course, we cannot describe the molecular differentiation model of CAFs explicitly at present, but through the development of pseudotime analysis, subgroup similarity analysis and RNA velocity analysis, we have made a great breakthrough in exploring the cellular molecular household model. For example, scRNA‐seq was integrated to identify the regulatory dynamics of the differentiation of human postpartum thymic transplanted progenitor cells and immature thymocytes,^81^ as well as the state study during the process of differentiation of the transcriptional map of lineage tracing,^82^ thus demonstrating the feasibility of this study. Although this finding has not been verified in any of the many systemic cancers in the body, the growing maturity of analytical techniques provides strong support for further research. Notably, CAFs can interact with stromal cells through cell–cell contact and cytokine release. For example, in prostate cancer, CAFs secrete CXCL14 and other related factors to promote macrophage M2 polarization. At the same time, tumour‐associated macrophages (TAMs) with M2 phenotype can activate CAFs and promote tumour progression.^83^, ^84^ In addition, several studies have revealed differentiation processes between different CAFs, but there are still many challenges and uncertainties in the study of the interaction between different CAF subtypes. Fortunately, based on single‐cell sequencing data combined with cell communication analysis, we can effectively demonstrate the interactions between different cell populations in the tumour microenvironment, as well as the mediated synergistic or inhibitory effect and develop novel anti‐tumour strategies accordingly.

### 4.2 New look for CAFs heterogeneity

**FIGURE 4:**
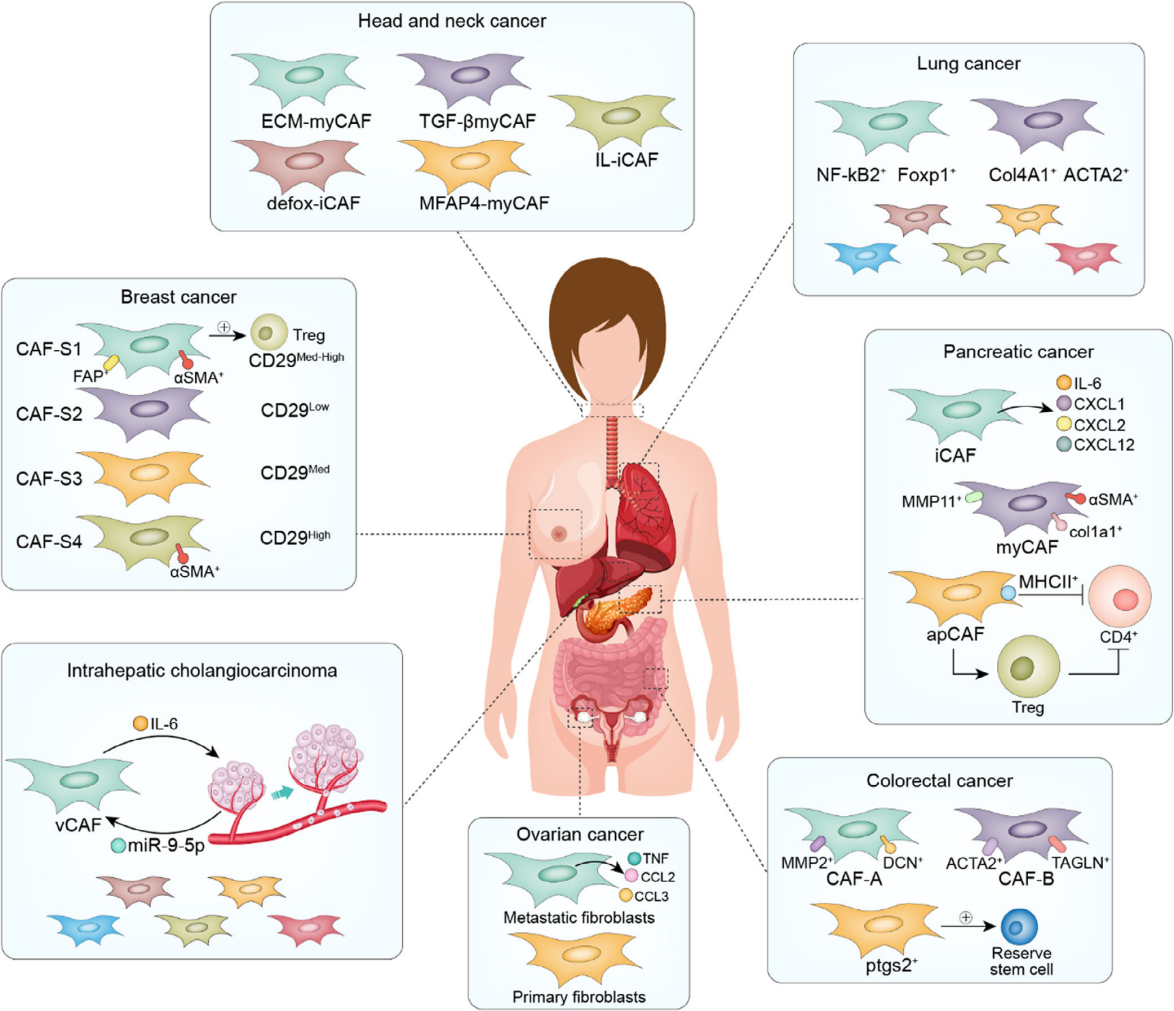
CAF subtypes in various cancer types. Various subtypes of iCAF and MyCAF are present in head and neck cancer, lung cancer and pancreatic ductal carcinoma, and apCAF is also present in PDAC, which can mediate immunosuppression. CAF‐S1 subtype in breast cancer mediates immunosuppression. There are two subtypes of CAF‐A and CAF‐B in colorectal cancer, and fibroblasts of PTGS2^+^ are closely related to reserving stem cells. In ovarian cancer, metastatic fibroblasts have more secretory functions than primary fibroblasts. In intrahepatic cholangiocarcinoma, the vCAF subtype promotes the malignancy of tumour cells by secreting IL‐6, and this process is positively fed back by the miR‐9‐5p secreted by tumour cells.

Due to the heavy workload of single‐cell sequencing analysis in the experimental stage and the strong heterogeneity of CAFs, it is difficult to analyse several types of cancers together at the beginning. Therefore, we will focus on the cancer types that have made breakthroughs in CAF research and classify and analyse them to find the potential rules (Figure 4).

#### 4.2.1 PDAC

Ohlund et al. first discovered the presence of two CAF subtypes, iCAFs and myCAFs in PDAC.^27^ The two CAFs showed unique transcription profiles through single‐cell sequencing. Compared with iCAFs, the *α‐SMA*, *TGF‐β* and *Col1a1* genes in myCAFs were significantly upregulated, while cytokines (such as IL‐6, IL11 and LIF) and chemokines (such as CXCL1 and CXCL2) in iCAFs were upregulated. Following experiments by Biffi et al. revealed different activatedsignaling pathways in iCAFs and myCAFs.^85^ IL‐1 involved in the formation of iCAFs, and TGFβ mediated the formation of myCAFs. Spatially, iCAFs are located in the tumour‐free region of the tumour and far away from the cancer cells and play a more important role in the deterioration, multidrug resistance and proliferation of PDAC than myCAFs. As previously mentioned, iCAFs secrete a large number of cytokines, among which IL‐6 promotes the progression of cancer,^86^, ^87^, ^88^ cachexia and immunosuppression.^89^ iCAFs specifically synthesize hyaluronic acid synthase (HAS1/HAS2).^38^ Extensive amounts of hyaluronic acid can cause drug resistance in cancer cells.^90^ Hypoxic regulator HIF1, REDOX regulator NRF2 (NFE2L2) and superoxide dismutase 2 (SOD2) are active in iCAFs, suggesting that iCAFs play a role in the remission of oxidative stress. myCAF have multiple functions, including contractility and matrix remodelling ability, smooth muscle contraction, focal adhesion and the formation of collagen. myCAFs are characterized by shrinkage protein gel (TAGLN), myosin light chain (MYL9), myosin 1 and 2 (TPM1, TPM2), matrix metallopeptidase 11 (MMP11), periostin (POSTN) and hox transcription factor (HOPX).

Other ScRNA‐seq studies subsequently identified different CAF subtypes. The FB1 subtype has a similar transcription profile with the iCAF phenotype described previously and is rich in the expression of cytokines and chemokines, including IL‐6, CXCL12 and CCL2. The FB3 subtype share characteristics of myCAFs, including the expression of ACTA2 and many myofibroblast‐associated contraction factor genes.^91^ Furthermore, subtypes B and D have the characteristics of myCAF in the expression of ACTA2 and many ECM components.^92^ Subtype C is somewhat similar to the iCAF phenotype in expressing inflammatory mediators such as complement components. Subtype A has the characteristics of iCAFs and myCAFs: the expression of ACTA2 is low, while the expression of myofibroblast genes such as POSTN is high. The subsequent study by Elyada E and others found some other CAF subtypes. The premise was consistent with the overall background of iCAFs and myCAFs determined by sequencing single cells across species, not only the analysis of the definition of iCAFs and myCAFs confirmed before.^38^ Besides, leucine‐rich repeat‐containing 15 (LRRC15) expressed myCAF‐like subpopulations were also identified in PDAC and negatively associated with anti‐programmed death ligand 1 (PD‐L1) therapy, demonstrating the immunosuppressive role of certain myCAFs.^35^ Recent studies reported the existence of an antigen‐presenting type of CAFs apCAFs. Apart from classic CAF‐ related pan‐fibroblast markers, such as PNPD and DCN, apCAFs highly express major histocompatibility complex (MHC) II class family gene, serum amyloid A3 (Saa3), a tumour‐promoting factor for pancreatic CAFs and leukocyte‐secreting peptidase inhibitors (Slpis). Pathway enrichment analysis of the apCAF genes demonstrated that the specific upregulation of the signalling pathways of fatty acid metabolism, mTORC1 signal transduction, MYC targets and, more importantly, antigen presentation and processing are observed. Functionally, MHC II genes H2‐A1 and CD74 are among the most active proteins in apCAFs, which can lead to a significant increase in the activity of other immune adjustment factors. Accordingly, apCAFs can induce CD4^+^ T cells through antigen presentation to a certain extent to reduce their activity, and can also mediate CD4^+^ T cell function through the inhibition of Tregs, thus reducing antitumor immunity. Therefore, although apCAFs have the function of antigen presentation, it is not consistent with normal antigen presentation activation. At the same time, apCAFs have differentiation abilities and can differentiate into iCAFs and myCAFs.

#### 4.2.2 Lung cancer

In the experiment of Diether Lambrechts et al,^93^ CAFs were divided into seven subtypes, among which cluster 1 is highly enriched in tumour cells, has a strong EMT transformation signal and TGF‐β gene expression, and has high expression of genes regulated by HOXB2 and FOXO1. Therefore, cluster 1 is an extracellular fibroblast matrix phenotype. At the same time, cluster 1 has slightly strong hypoxic‐related genes and mitotic spindles, indicating that cluster 1 has strong fission ability while inducing hypoxia. Compared with other subpopulations, ACTA2 of cluster 2 is highly expressed MEF2C and its target genes, while the expression of genes regulated by MSC is low. Given that MEF2C is a myogenic agonist^94^ and MSC a myogenic antagonist,^95^ cluster 2 is considered as a possible candidate for myogenesis. The other subtypes have specific collagen fibres expressed in their subtypes, and cluster 6 is enriched in nonmalignant tumours. Cluster 7 is not expressed in non‐small‐cell lung cancer (NSCLC), but TNF‐α signalling via NF‐κB‐related genes is highly expressed in cluster 7.

#### 4.2.3 Colorectal cancer

CAF‐A and CAF‐B subtypes were identified in colorectal cancers.^51^ CAF‐A highly expressed in MMP2, DCN and COL1A2, while CAF‐B highly expressed in ACTA2, TAGLN and PDGFA. In another experiment, fibroblasts were divided into six clusters (cluster 0–5) with high FAP expression, among which clusters 1, 2 and 4 were fibroblast‐like cells with high expression of DCN; clusters 0 and 3 were smooth‐muscle‐like cells with high expression of ACTA2; and cluster 5 was pericytes, where the angiogenic genes notch3 and angpt2 were highly expressed.^96^ More recently, scRNA‐seq identified a rare set of fibroblasts expressing ptgs2, which made peanut tetraenoic acid into highly variable prostaglandin e2 (PGE2).^97^ Fibroblasts‐derived PGE2 triggers the expansion of sca‐1 stockpile stem cell group and this process is dependent on Hippo pathway transcriptional effector YAP. In another report, based on the tumour heterogeneity landscape analysis in colorectal cancer, the functions of two CAFs subpopulations were identified, mCAFs is associated with myofibroblast‐like cells, and iCAFs is associated with immune inflammation. Besides, there is extensive crosstalk between iCAFs and stromal components in the TME to promote tumour progression. At the same time, some anti‐tumour immune cells such as NK cells were significantly reduced in iCAFs‐enriched cluster.^98^

#### 4.2.4 Ovarian cancer

In a single‐cell analysis of human ovarian tumours uncovered two main subclusters of CAFs, defined as TGFB CAF, which displayed high myCAF and TGFB CAF gene profiles, and IL1 CAF, which predominantly showed iCAF and IL1 CAF signature expression as previously described in PDAC.^35^, ^38^, ^99^ TGFB CAF expresses genes correlated with TGF‐β‐induced reactive stroma, and POSTN, ACTA2, collagens (COL10A1, COL11A1), MMP11 and FN1, whereas IL1 CAF upregulated genes associated with cytokine/chemokine signalling features, including CXCL14 and CCL2.^99^ Another study using ovarian cancer samples from patients with primary or metastatic sites identified two subsets of CAFs annotated as primary fibroblasts and metastatic fibroblasts. In comparison with primary fibroblasts, metastatic fibroblasts express higher levels of soluble factors, including IL‐6, and CXCL12, suggesting the potential of metastatic fibroblasts to produce secreted factor to create a favourable microenvironment for tumour cell invasion. Interestingly, The primary and metastatic fibroblasts marked by a striking upregulation of collagen genes, MMP and MMP‐associated genes, indicative of a plausible role for ECM remodelling in tumour development.^100^

#### 4.2.5 Head and neck cancer

The first scRNA‐seq of human head and neck squamous cell carcinoma (HNSCC) identified three distinct CAF subpopulations: myCAFs, expressing high levels of ACTA2 and MYL9, classic CAFs, marked by receptor and ECM‐related genes, including FAP, PDPN and CTGF, or resting CAFs, which was lack of myCAF and classic CAF markers.^101^ Although the respective functional roles are still not clear, these TGF‐β^+^ CAFs are tightly associated with tumour invasion and adverse pathologic features by promoting the partial EMT at tumour edge in paracrine manner. In another study, one using human papillomavirus (HPV)^+^ and HPV^−^ patient derived HNSCC specimens, the relevant fibroblasts were separated into three sub‐states including normal fibroblasts (NAF), classic CAFs, or innovative elastic CAFs according to their gene expressions.^102^ Importantly, elastic CAFs with increased express elastic fibre differentiation genes such as microfibril associated protein 4 (MFAP4), function as a negative prognostic factor in HPV^+^, indicative of therapeutically targetable. Recently, Young J. Kim's group using scRNA‐seq from four patient head and neck tumours revealed obviously changing CAF subtypes after aPD‐1 therapy. Among them, CAF‐0/3 function as positive predictors of aPD‐1 response, whereas CAF‐1 promotes immunosuppression.^103^ In spite of differences in CAF classifications that may result from distinct models and taxonomy, these reports highlight the heterogeneity and functions of CAFs in shaping HNSCC microenvironment and resistance.

#### 4.2.6 Bladder urothelial carcinoma

scRNA‐seq profiling of human bladder urothelial carcinomas identified two subsets of CAFs, referred to as iCAFs and mCAFs. The iCAFs (PDGFRA^+^) upregulated inflammatory cytokines and chemokines, such as IL6 and CXCL12, and mCAFs (RGS5^+^) enriched in ECM‐associated pathways, which all resemble iCAFs in PDAC, suggesting analogous CAF subgroups, at least in part, across caner types.^27^, ^104^ In particular, iCAFs have pro‐proliferation functions and were the dominant factors to promote bladder urothelial carcinoma progression.^104^

#### 4.2.7 Intrahepatic cholangiocarcinoma

In a recent study on human intrahepatic cholangiocarcinoma, six subtypes of CAFs were identified. Vascular CAFs (vCAFs) have high expression of microvascular‐characterized genes (CD146, GJA4, MYH11 and RGS5) and inflammatory chemokines (IL‐6, CXCL12) and are significantly enriched in genes related to hypoxia response and mesenchymal cell proliferation. The matrix CAF (mCAF) has low expression of α‐SMA and high expression of extracellular matrix (COL6A3, Fn1, Lum, POSTN, COL5A2, COL5A1, DCN). Additionally, inflammatory CAFs (iCAFs) had low α‐SMA expression but high CXCL1, SLPI, IGFBP6, FBLN1, IGFI, SAA1 and complement genes (C3 and C7). There is also a subtype similar to apCAFs in PDAC. The remaining two subtypes are EMT‐like CAFs (eCAFs) and lipofibroblasts. Moreover, vCAFs promoted the occurrence and development of intrahepatic cholangiocarcinoma through the IL‐6/IL‐6R signalling pathway and promoted the dry nature of tumour cells.^105^

#### 4.2.8 Breast cancer

Ana Costa et al. studied breast cancer in detail. It was concluded that the four kinds of CAF subtypes.^106^ A CAF marker‐intensity distribution‐based decision tree found different CAFs in different pathological types of breast cancer and different proportions of the subtypes of CAFs. CAF‐S1 and CAF‐S4 fibroblasts are characterized by the regulation of the actin cytoskeleton, muscle contraction and oxidative metabolism. Compared with CAF‐S4, CAF‐S1 was more enriched in immune signals (including cytokine production and regulatory T cells (Tregs). Therefore, by comparing previous studies, the authors determined the list of CAF‐S1 genes known to be related to immune regulation.^107^, ^108^, ^109^, ^110^, ^111^ These include chemokine signal transduction (CXCL12, CXCL13, CXCL14 and CCL11), cell adhesion (JAM2) and immunomodulatory function (PDCD1LG2/PD‐L2, TNFSF4/OX40L, DPP4, CD276/B7H3 and NT5E/CD73). It was further verified that CAF‐S1 mediated antitumor immunity. However, combined with previous studies, we also wanted to find an association between the CAF subtype of breast cancer and iCAFs and myCAFs. According to the latest study by Yann Kieffer,^112^ through individual sequencing analysis of the CAF‐S1 subtype, we obtained eight different subtypes (Cluster 0‐Cluster 7), with each subtype having its own marker. Most importantly, we combine the CAF subtype in BC with PDAC. Cluster 1, Cluster 2 and Cluster 5 belong to the iCAF group; Cluster 5 corresponds to the apCAF subgroup; and Cluster 3, Cluster 4, Cluster 6 and Cluster 7 belong to the myCAF subgroup.

Ana Costa et al. further explored CAFs in metastatic lymph nodes of breast cancer.^113^ Through single‐cell sequencing and immunohistochemical analysis, it was found that CAFs in lymph node metastasis were highly similar to the CAFs of breast cancer and could be divided into four subtypes (CAF S1, CAF S2, CAF S3, CAF S4). Based on unsupervised principal component analysis (PCA) and hierarchical clustering (IHC), transcriptome differences of the CAF subset were visualized. Combined with paired difference analysis, it was found that the upregulated genes of CAF‐S1 were mainly involved in ECM tissue formation, while the specific genes of CAF‐S4 were involved in muscle contraction. It is not difficult to determine that the CAF classification and function of primary and metastatic CAFs are similar. Then, an on‐plate tumour system and inverted Transwell technology were used to comprehensively analyse the occurrence of metastasis and invasion. CAF‐S1 secreted factors attracting cancer cells, and CAF‐S1 initiated the first step of EMT, while CAF‐S4 remodelled the stroma and promoted 3D cell invasion in cancer. In terms of mechanism, further investigation revealed that CAF‐S1 fibroblasts promoted BC cell migration and EMT initiation in cancer cells in a CXCL12‐ and TGF β‐dependent manner. In addition, caf‐s4 fibroblasts can stimulate 3D cell invasion and movement by increasing the contraction force of the NOTCH signalling pathway. However, CAF S2 and CAF S4 subtypes have been defined as fibroblast populations in previous studies on breast cancer and lymph node metastasis and perform the most basic roles of fibroblasts in the process of tumour development.

Since breast cancer immunotherapy has been developed and improved, there have been more studies on CAFs. In Michael Bartoschek's study in humans,^114^ CAFs in breast cancer were analysed at the single‐cell level and then through four different types of CAF gene expression. vCAFs were enriched during blood vessel growth with angiogenesis genes, and vCAF subtypes originated in the week of vascular cell growth and then in the process of tumour progression into the tumour stroma. The genetic characteristics of vCAFs and collected data from case–control studies of endothelial cell genes and microvascular system features are closely related.^115^, ^116^ Genes with significant differences in vCAFs include vascular regulators such as Notch3, Nr2f2, Epas1 and Col18a1. mCAFs were enriched in EMT and ECM‐related genes, originated from normal fibroblasts of the original tissue, and were highly correlated with stromal‐derived invasion characteristics and stromal‐related therapeutic prediction characteristics.^117^, ^118^ The mCAF subset specifically expressed transcripts of a variety ofECM‐related genes, such as glycoproteins (Lum, DCN and Vcan), stromal proteins (Smoc, Fbln2 and Fbln1), structural proteins (Col4a1) and matrix‐modifying enzymes (Lox and Loxl1). In addition, mCAFs expressed the immune cell attractant CXCL14 in large quantities, suggesting their role in regulating the tumour immune response. cCAFs are the proliferating segment of vCAFs. cCAFs are mostly concentrated in the division phase. Finally, dCAFs are related to cell differentiation and tissue development and morphogenesis, and they may be derived from tumour cells undergoing epithelial‐mesenchymal transformation (EMT) and express CD10/Gpr77 and Hedgehog target genes, thereby promoting the characteristics of cancer stem cells.^119^, ^120^ dCAFs specifically express CD10 and Gpr77, thus increasing the possibility of malignant stem cells to maintain their own niche through EMT.

#### 4.2.9 pan‐cancer

It is worth noting that some reports perform systematic investigations on their ubiquitous characteristics across different cancer types. They mainly provided a comprehensive overview of the common features and dynamics of CAFs and highlights their heterogeneity in different tumours. Some reports revealed that the activation trajectory of the major CAF subtypes was significantly different, which showed obvious interactions with other cellular components.^121^ Moreover, CAFs may exert immunosuppressive effects in both pan‐cancer and ovarian cancer, which may explain accelerated tumour progression and poor outcomes based on the multi‐omic data at the pan‐cancer level.^122^

In summary, when we enumerate the CAF subgroups in the TME of each system. It is not difficult to find that although CAF is highly heterogeneous, different teams also have certain differences in the definition of CAF. The different activated CAF subsets manage to adapt to the tumour microenvironment and interact with tumour cells, resulting in a variety of malignant tumour behaviours via paracrine signalling. Different CAF subsets also show a certain degree of heterogeneity in secretory phenotypes. They exert different effects in tumour microenvironment by secreting unique cytokines and chemokines, such as tumorigenesis, tumour angiogenesis, tumour metastasis and drug resistance.^27^ However, iCAF and MyCAF have always been the core functional subsets in CAF, and these two subsets also mediate the main role of regulating tumour invasion, metastasis and immune escape. In the aspects of clinical values, FAP is a cell surface marker for these CAF subtypes in over 90% of human cancers, vaccination against the FAP antigen carried by these CAFs may be the ideal strategy for cancer immunotherapy.^123^

## 5 CLINICAL APPLICATION OF SINGLE‐CELL RNA SEQUENCING IN CAFS

Targeted treatment around the core functional subsets of CAF summarized above is one of the most significant things that single cell sequencing has brought to us. Here, we summarized the applications of single‐cell sequencing in different tissues (Table 2).

**TABLE 2:** The applications of single‐cell sequencing.

Tissues	Species	Major results	References
Brain	Mouse	Analysis was performed to identify five different transcriptome astrocyte subtypes in the cortex and hippocampus	^79^
Chronic inflammation	Mouse	Distinct subpopulations of fibroblasts with different cytokine expressions and signals were identified in the wounds of old mice with slow versus fast healing rates	^131^
Lung	Human	A single‐cell atlas of pulmonary fibrosis was generated. Using this atlas, heterogeneity within alveolar macrophages and epithelial cells from subjects with pulmonary fibrosis were demonstrated	^132^
Kidney and skin	Human	Dissecting the molecular heterogeneity in lupus nephritis	^133^
Colorectal cancer	Mouse/Human	Six groups of tumours infiltrating Innate lymphoid cells (ILCs) with unique characteristics were found	^134^
Lung	Mouse/Human	By studying the effect of KRAS (G12C) inhibitor treatment at single‐cell resolution, response rates were testedin the same gene cell population	^135^
Liver	Human	Outlining the characteristics of resident cells and providing a map of the human hepatic immune microenvironment	^136^
Immunity (spleen and blood)	Human and mouse	Identifying organ‐specific signatures and conserved NK cell subsets in humans and mice	^137^

Prior to single‐cell sequencing, the cell heterogeneity was often ignored, causing great trouble. For example, in pancreatic cancer, different therapeutic approaches targeting CAFs have obtained conflicting and sometimes even worse results.^67^, ^69^, ^124^ Moreover, attempts to deplete CAFs based on α‐SMA expression resulted in reduced survival in mice.^67^ Through single‐cell sequencing analysis of the CAFs population in pancreatic cancer, it is not difficult to see that the iCAF secretion phenotype indicates that this CAF subtype plays a major role in the invasion and progression of pancreatic cancer. Although myCAFs play a certain role in pancreatic cancer and the content of myCAFs increases significantly with the progression of cancer in general, their function is less than that of iCAFs. Through the analysis of previous treatment regimens, it is not difficult to find that therapy targeting at CAF population without distinction may lead to malignant outcomes,^125^, ^126^, ^127^, ^128^ which may result from the heterogeneity and plasticity of CAFs. On the one hand, myCAFs have physical effects that can wrap tumour tissues and prevent tumour invasion, thus displaying tumour‐restraining function in some tumours. Besides, iCAFs and myCAFs can be transformed into each other in some circumstances. The extensive inhibition of myCAFs leads to a relative increase in iCAFs. Therefore, in the case of pancreatic cancer, we can target iCAFs. The benefits of targeting iCAFs include the number of iCAFs and thus the production of pretumor cytokines or chemokines, and alleviating transformation of iCAFs into myCAF.^67^, ^124^ Indeed, JAK/STAT3 signalling pathway inhibitor, which suppresses iCAF activity and function, significantly increases the myCAF/iCAF ratio in tumour therapy.^129^, ^130^ In addition, single‐cell sequencing identified angiotensin receptor type II as an underlying marker of iCAF subpopulation. Therefore, angiotensin II inhibitors (losartan), can also suppress iCAFs.^138^, ^139^

During the cancer treatment process, tumour cells may escape immune cell attention, partly by tricking CD4^+^ T cells or by Treg cell inhibition of CD4^+^ T cells. Latest research on apCAFs revealed their immunosuppressive functions by activating Treg. Since apCAFs have differentiation potential, it is feasible to convert them into myCAFs to reduce their functions.

Breast cancer has also been thoroughly studied in CAFs due to the effectiveness of immunotherapy. But while immunotherapy can be effective against some tumours, it is not clear why it does not work in some patients.^106^ Among the four CAF subtypes obtained by the combination of flow cytometry and single‐cell sequencing, CAF‐S1 has a strong immunosuppression effect. CAF‐S1 can promote the enrichment of CD4^+^CD25^+^ T cells and guide their transformation into CD25^+^FOXP3^+^ Treg cells. DPP4 is the key to CAF‐S1‐mediated regulation of Treg cells, hence immunotherapy can be accomplished to a certain extent by inhibiting the role of DPP4. Furtherly, subsequent study suggests that subpopulations expressing ECM and TGFβ signalling in CAF‐S1 were the key to mediate primary resistance to immunotherapies.^112^

In human intrahepatic cholangiocarcinoma,^105^ vascular‐derived vCAFs have been found to be strongly associated with tumour cells through the IL‐6/IL‐6R axis, which not only promote the occurrence of tumours but also further mediate their progression and invasion. Targeting vCAFs or inhibit the IL‐6/IL‐6R axis in tumour cells would be an effective way to reduce the degree of malignancy.

In a comprehensive view of CAF‐subtype‐directed cancer treatment, first, CAF subtypes are classified into iCAFs and myCAFs. In different cancer types, and even in different treatment schemes, we need to target the appropriate CAF subtypes. This is a further indication of the importance of single‐cell sequencing because it provides a detailed subgroup classification as well as insight into the possible functions of each subtype. In this way, the cancer type and the function of the CAF subgroup can be combined to select the appropriate treatment.

## 6 CONCLUSIONS AND PERSPECTIVES

From the research on CAF subtypes by single‐cell sequencing in recent years, it is not hard to see that with the rapid development of single‐cell sequencing technology, the classification of CAF subtypes can be revealed to a large extent. Therefore, we proposed a bold hypothesis to envision the targeting therapy of specific CAF subtypes.

Firstly, we can explicitly define different CAF subtypes and their various markers. Second, the advent of protein groups allows us to understand the situation of CAF subtypes. The cellular indexing of transcriptomes and epitopes by sequencing (CITE‐seq), which combines measurement of RNA and protein expression, was also developed. At the same time, the genetic expression of gene expression and tissue slices is integrated so that the gene expression of different cells in the tissue is located in the original space of the organization. The difference between the genes in the organization is the difference between the genes in the organization.^140^ Therefore, these new technologies will provide more precise help for our subtype classification.

In addition, cytokines and chemokines secreted by some CAF populations can lead to increased chemotherapy resistance and invasiveness of tumours.^141^ Multidrug resistance mediated by these chemokines, such as CXCL12, in tumours has been widely reported.^142^, ^143^ The distribution of cytokines secreted by tumours was analysed. It was found that CAF‐S1 was significantly correlated with interleukin‐17fF (IL‐17F), IL‐1B, IL‐10 and IL‐6. CD10 and GPR77 cells surface molecule‐positive fibroblasts were found. This CAF subgroup promoted chemical resistance and cancer formation in breast and lung cancer patients. CD10^+^GPR77^+^ CAFs secrete a large amount of interleukin‐IL‐6 and IL‐8, which provide a living environment for cancer stem cells (CSCs) through sustained NF‐κB signalling, while IL‐6 promote ER^+^ breast cancer cell proliferation, thus playing an important role in chemotherapy resistance in breast cancer cells.^144^, ^145^ In addition, IL‐6 has been shown to mediate resistance to paclitaxel and adriamycin in ER^+^ breast cancer and to trastuzumab in Her‐2‐positive tumours.^145^, ^146^ It is easy to find that in view of the CAF immunotherapy we paid much effort, that we can find guidance for CAF immunotherapy through the use of many markers. We can distinguish between the CAF subsets of a small part of the tumour, but these markers do not have strong specificity. We will not be able to go through these surface markers for CAF subgroup immune therapy. After subtypes were clearly classified by single‐cell sequencing technology, phenotypic differences, and protein expression differences of different CAFs could be observed in detail through single‐cell sequencing, and we could clearly locate the subtypes we wanted to target and thus achieve inhibition. Moreover, the combination of scRNA‐seq and spatial transcriptome and the combination of metabolomics can more accurately define the specificity of different CAF subsets in the analysis of CAF, providing strong early guidance for our targeted therapy.

Finally, in order to simply deplete CAFs to reduce their adverse effects, researchers have begun to study on reprogramming CAFs into normal fibroblasts. For example, to prevent the role of the activation of pancreatic stellate cells through their abnormal expression of heat shock protein 47 (HSP47) as a target, and tumour tissues have a higher expression of HSP47 than normal tissues, a nanometre system for pH was designed; combined with ATRA and HSP47 siRNA activity, not only did PSCs transition into static‐state PSCs, but the production of ECM was also reduced in vivo and in vitro.^147^ Moreover, ATRA can enhance T cell infiltration and promote antitumor immunity.^148^ Changes in the active state of CAFs can also be achieved through vitamin D or calcitriol. The stimulation of vitamin D receptors can also achieve the same effect.^149^ However, we found that we only drew conclusions in response to the phenomenon. When we revealed the molecular differentiation process of CAFs through single‐cell sequencing technology, the targeted reprogramming of CAF subgroups to induce cancer progression could achieve the maximum efficiency of this method.

In conclusion, the emerging approach of single‐cell sequencing provides a great impetus for us to explore the heterogeneity of CAFs. We summarized certain rules of the existence of CAF subtypes, and these findings can have a strong guiding role for clinical treatment base on previous research.

## AUTHOR CONTRIBUTIONS

Xiangjian Zhang, Ruiqiu Zhu and Die Yu have contributed equally to the writing process of this manuscript. Ke Xu designed and guided the review. Xiangjian Zhang, Ruiqiu Zhu and Die Yu and Ke Xu wrote and edited the manuscript. Juan Wang, Yuxiang Yan helped with reference collection and draw the figures. All authors read and approved the final manuscript.

## CONFLICT OF INTEREST STATEMENT

The authors declare no competing interests.
